# Preload and Removal Torque of Two Different Prosthetic Screw Coatings—A Laboratory Study

**DOI:** 10.3390/ma17061414

**Published:** 2024-03-20

**Authors:** Lara Coelho, José Manuel Mendes, Joana Mendes, Carlos Aroso, António Sérgio Silva, Maria-Cristina Manzanares-Céspedes

**Affiliations:** 1 (UNIPRO)—Oral Pathology and Rehabilitation Research Unit, University Institute of Health Sciences (IUCS), CESPU, 4585-116 Gandra, Portugal; lara.coelho@iucs.cespu.pt (L.C.); joana.silva.mendes@iucs.cespu.pt (J.M.); carlos.ribeiro@iucs.cespu.pt (C.A.); asergio.silva@iucs.cespu.pt (A.S.S.); mcmanzanares@ub.edu (M.-C.M.-C.); 2 Human Anatomy and Embryology Unit, Faculty of Medicine and Health Sciences, University of Barcelona, 08007 Barcelona, Spain

**Keywords:** dental implant single tooth, torque, lubricants, bone screw, dental abutments, silicones, biocompatible coated materials

## Abstract

This study aimed to evaluate the effect of two coating materials, a silicone sealing gel and a polytetrafluoroethylene (PTFE) tape, on the screw preload and removal torque value (RTV) to develop strategies to prevent prosthetic screw loosening. We examined 45 complexes comprising an implant, abutment, and prosthetic screw, of which 15 samples were uncoated, 15 were coated with GapSeal^®^ (Hager & Werken GmbH & Co., Duisburg, Germany), and 15 were coated with PTFE tape (MIARCO^®^, Valencia, Spain). The screws were tightened to register the preload and then untightened to register the RTV. The preload values showed a statistically significant difference only in the PTFE group, suggesting that this lubricant negatively affects the preload. The RTVs showed statistically significant differences among all groups, with the GapSeal^®^ group and PTFE group showing the highest and lowest values, respectively. It can be concluded that the application of the PTFE tape on the screw significantly reduced the preload and RTV. The silicone sealing gel did not affect the preload but increased the RTV. Therefore, the use of GapSeal^®^ should be considered to prevent prosthetic screw loosening, while the use of PTFE tape should be avoided.

## 1. Introduction

Currently, dental implant-supported rehabilitation is the best option for replacing missing teeth and improving the quality of life of patients [1,2,3]. Nowadays, partially edentulous patients represent the main group of candidates for oral rehabilitation with implants. However, despite the high success rates, some biological and mechanical complications have been reported, with the loosening of the prosthetic screw being the most commonly cited [4,5,6]. In the event of screw loosening, the crown shows instability and micromotion, which can cause irritation to the soft tissues around the implant, pain, an unpleasant taste, and even implant fracture, compromising the implant’s survival rate [7,8].

It is important to understand the mechanics of the used screw to know how to prevent it from loosening. The prosthetic screw is intended to join the implant with the prosthetic abutment functionally. Thus, the abutment is screwed in by applying a torque that generates a series of forces in the abutment–implant complex, called a preload, which keeps the threads of the screw connected to their counterparts in the implant and keeps the components together [9,10].

The amount of preload on the threads of the prosthetic screw depends on the torque applied, the presence of materials or substances acting as lubricants, the physical properties of the materials, and the settling effect [11].

The settling effect plays a critical role in the stability of the screw. It has been reported that approximately 2–10% of the initial preload is lost. This could be attributed to the screw surface not being completely smooth, and because of this microroughness, the screw and implant are not completely in contact. Settling occurs when the rough spots flatten out. Thread friction is greater in the first adjustment and decreases with subsequent repetitions; hence, some authors recommend re-tightening the screw after 10 min [12].

Siamos et al. presented an interesting finding that increasing the removal torque value (RTV) is necessary when the initial applied torque is increased [9]. Modifications to the prosthetic screw have been proposed to promote greater preload stability. Moreover, several studies have evaluated ways to increase the preload without increasing the torque by reducing the coefficient of friction using various coating materials already applied on the screw and sold by the manufacturers such as pure gold, tungsten carbide, and diamond-like carbon [13,14]. However, some screws come loose after placement and function, resulting in the need to evaluate coating materials that could be applied during dental appointments. Some compounds, such as saliva (human or artificial), blood, Vaseline, chlorhexidine (CHX), and fluoride, have been extensively studied for their lubricating action [15,16]. Other studies evaluated the application of a bonding agent such as Loctite^®^, obtaining excellent results. However, its application in living human beings is unknown in terms of biocompatibility and cytotoxicity, so this recommendation is dubious [17,18,19].

In implantology, products have been developed that provide a protective seal on the empty spaces in implants. These products are mainly used to prevent peri-implantitis, being useful against bacteria, viruses, and fungi. They are viscous materials that never harden, thus making an airtight seal of the open spaces. They are hydrophobic and can be removed with hydrogen peroxide and alcohol. The use of these products to prevent unscrewing is beginning to gain popularity, although the preliminary results are contradictory [20].

Another study tested the applicability of polytetrafluoroethylene (PTFE) tape as a coating material. This material is used in medical devices since it has good biocompatibility, is resistant to chemical agents, and is hydrophobic. It is also adaptable and has good durability and thermal and chemical stability. Results obtained on PTFE’s use as an alternative coating material to avoid the loss of the prosthetic abutment screw are favorable [21].

Few articles have tested the applicability of these materials as screw coatings in order to prevent prosthetic screws from loosening. Therefore, we consider it important to test their behavior, as these coating materials could be a solution to one of the most frequent mechanical complications following the placement of implant-supported single crowns.

In this study, we aimed to evaluate the effect of two coating materials, a silicone sealing gel and a PTFE tape, on the screw preload and RTV. We also evaluated the relationship between the preload and the RTV. The null hypothesis was that the sealing gel and PTFE tape do not affect the screw preload and RTV and there is no relationship between the preload and the RTV.

## 2. Materials and Methods

A standard laboratory protocol was established and applied to test all selected samples at the Laboratory of Investigation in Oral Rehabilitation and Prosthodontics, UNIPRO—Oral Pathology and Rehabilitation Research Unit, University Institute of Health Sciences, Cooperative for Polytechnic and University Education, (IUCS), CESPU Gandra, Portugal.

### 2.1. Sample Size and Preparation

The sample size calculated for each group was 14, considering a minimum effect size of 0.5, a power of β = 0.80, and a significance of α = 0.05.

This study examined 45 complexes composed of 45 external hexagonal implants (length, 11.5 mm; diameter, 4.0 mm), 45 straight hexagonal abutments (cuff height, 1 mm; length, 7 mm), and 45 original titanium prosthetic screws (1.7 Torx). All components were obtained from DIU^®^ IMPLANT, Busan, Republic of Korea.

The abutments were first connected to the implants and equally divided into three groups. The control group (GC; *n* = 15) comprised samples without any coating. In the GapSeal^®^ group (GG; *n* = 15), a biologically safe sealing agent, GapSeal^®^ (Lot: 01/14; Hager & Werken GmbH & Co., Duisburg, Germany), was injected into the internal compartment of the implants. GapSeal^®^ is composed of a silicone matrix and thymol antibacterial agent and is a highly viscous material that never hardens; thus, there are no shrinkage gaps as observed with curing substances. In the PTFE group (GP; *n* = 15), the abutment screws were wrapped with a PTFE tape (COD 269; 12 mm × 12 m × 0.075 mm; MIARCO^®^, Valencia, Spain) that was wound twice around the screws, and the PTFE tail at the bottom of the screw was then cut.

Initially, all screws were manually screwed and coded. All tests were performed by trained operators.

### 2.2. Measuring the Preload and RTV

The preload was measured using an advanced touchscreen force gauge Centor Touch Star TH^®^ (Andilog Technologies, Vitrolles, France) validated following the AB certification (ISO 9001:2015 [22]). The accessory for measuring the preload was tightened using an M5 thread at the bottom of the device. This accessory is similar to the chuck of a drilling machine and is connected to a device by a cable. The chuck retains the sample by tightening around it with its own key, similar to the retention of the spawn in a drill. Caligraph^®^ version 12.20 (Andilog Technologies, Vitrolles, France) was used for data acquisition. Data acquisition was continuously performed using a computer at a frequency of 1000 Hz. Therefore, the acquired data consisted of around 1000 measurement points every second.

**Figure 1:**
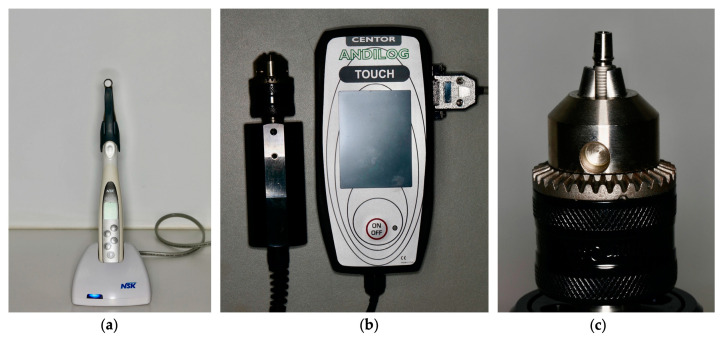
(**a**) NSK digital torque meter, (**b**) Centor Touch Star TH^®^ and (**c**) sample fixed in the Centor Touch Star TH^®^ device.

Each sample was fixed in the device (Figure 1), and the abutment screw was torqued at 30 N cm as per the manufacturer’s recommendation, using a screwdriver and digital torque meter (iSD900 with the CE0197; NSK^®^ Dental Spain SA, Madrid, Spain) that was calibrated after each measurement. When the screws were torqued, the force gauge was used to record the preload generated by the abutment screws.

After tightening the abutment screws in the three groups to 30 N cm and recording the preload, the screws were loosened to measure the RTV. The loosening torque of each sample was recorded.

### 2.3. Statistical Analysis

Data were analyzed using R, version 4.3.0 (R Core Team, 2023) [23]. Some of the libraries used included {car}, {ggplot2}, {ggpubr}, {multcomp}, and {effect size}. Descriptive statistics are presented as means (M) and standard deviations (SD) for univariate analysis and adjusted means (adjM) and standard errors (SE) for multivariate analysis. ANOVAs were used to compare the preload and RTVs among the groups. Analysis of covariance (ANCOVA) was used to compare the RTVs among the groups, adjusted to the baseline preload. Effect size was calculated as eta squared (η^2^) for ANOVAs and partial eta squared (η^2^_p_) for ANCOVA. The thresholds representing small, medium, and large effects were 0.01, 0.06, and 0.14, respectively. The Shapiro–Wilk test was used to assess the normality of the distributions, and statistical significance was set at *p* > 0.05. The Levene test was used to assess variance homogeneity, which was confirmed at *p* > 0.05. R^2^ was calculated to assess the variance of the outcomes explained by the explanatory variables. The significance threshold was set at *p* < 0.05.

## 3. Results

**Figure 2:**
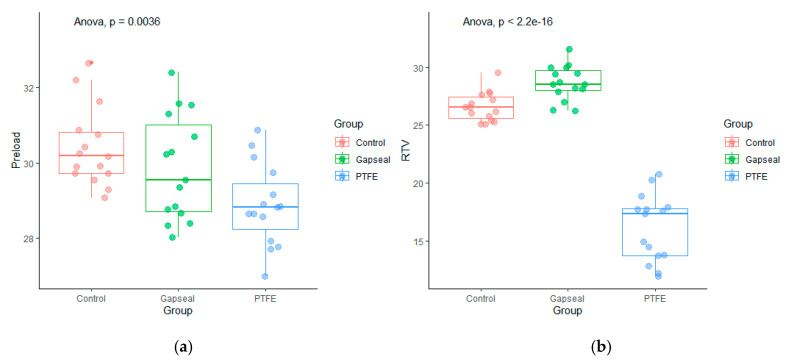
(**a**) Boxplot for preload comparisons by group and (**b**) Boxplot for RTV comparisons by group.

**Figure 3:**
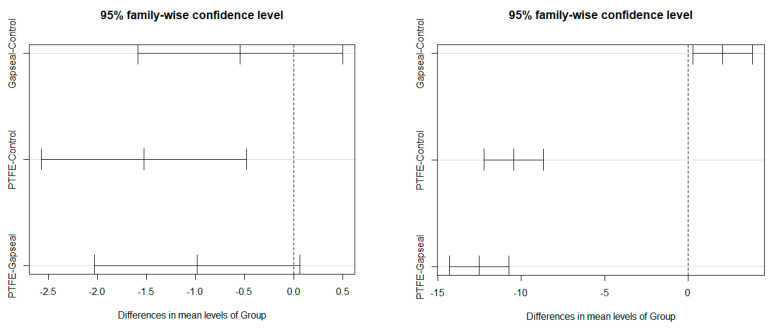
Tukey multiple comparisons tests for group comparisons of preload (**left**) and RTVs (**right**).

Table 1 shows a comparison of the preload and RTVs between the groups. Figure 2 shows the preload and RTV distribution in the groups. Figure 3 shows the Tukey multiple comparison test results for the group comparisons of the preload and RTVs.

The univariate group comparisons for preload revealed statistically significant differences, with a high effect size of F_(2,42)_ = 6.45 (*p* = 0.004) and η^2^ = 0.23. The Tukey multiple comparison test revealed a significantly higher preload in the GC group (M = 30.41, SD = 1.04) than in the GP group (M = 28.88, SD = 1.07) (*p* < 0.05). The R^2^ coefficient for the preload model explained by the group was 19.86%.

The univariate group comparisons for RTVs revealed statistically significant differences, with a high effect size of F_(2,42)_ = 167.00 (*p* < 0.001) and η^2^ = 0.83. The Tukey multiple comparisons test revealed significant differences in the RTVs between all the groups (*p* < 0.05). The highest RTV was identified in the GG group (M = 28.67, SD = 1.47), followed by the GC group (M = 26.59, SD = 1.25) and GP group (M = 16.15, SD = 2.89). The R^2^ coefficient for the RTV model explained by group was 88.30%.

Since a significant association was observed between preload and group, the RTV comparisons among the groups were adjusted to the preload using an ANCOVA. The ANCOVA results revealed statistically significant differences between groups, with a high effect size (F_(2,41)_ = 177.04 [*p* < 0.001]; η^2^_p_ = 0.90), and almost significant results for preload, with a moderate effect size (F_(1,41)_ = 3.53 [*p* = 0.068]; η^2^_p_ = 0.08). The Tukey multiple comparisons test revealed differences between all the groups (*p* < 0.05). The highest RTV was identified in the GG group (adjM = 28.70, SE = 0.49), followed by the GC group (adjM = 26.40, SE = 0.59) and GP group (adjM = 17.20, SE = 0.63). When compared with unadjusted means, the adjM values showed that the preload increased the RTV in the GG and GP groups and decreased it in the GC groups. The R^2^ coefficient for the RTV model explained by group + preload was 88.96%.

These results suggest that the RTV was significantly higher in the GG group than in the GC group, even when the baseline effect of the preload was considered. The GP group maintained the lowest results among the three groups, confirming the results obtained in the univariate analysis.

## 4. Discussion

### 4.1. Preload Analysis

The amount of preload on the prosthetic screw depends on the torque applied, the presence of a lubricant, the physical properties of the materials, and the settling effect [11]. The magnitude of settling depends on the initial surface roughness, the surface hardness, and the magnitude of the loading forces. Rough surfaces lead to a higher friction coefficient and increased settling, thereby decreasing the preload [9].

Assuming that obtaining a high preload will prevent the prosthetic abutment screw from loosening, this study investigated the influence of using a PTFE tape wrapping or silicone sealing gel on the prosthetic screws of implant-supported single crowns on the preload and RTV.

The results of the preload statistical analysis revealed significant differences only between the GC and GP groups, while the GG group presented results quite similar to those of the GC group. Therefore, the null hypothesis was rejected for the group using PTFE tape as a lubricant and accepted for the group using the sealing silicone gel.

To our knowledge, only one study [14] has evaluated the effect of sealing silicone gel on the preload, and their results are similar to ours. The authors tested four different lubricants, of which one was a sealing silicone gel, and they concluded that the investigated lubrication agents did not influence the preload when compared with the dry control. Only two samples showed a statistically significant difference; however, they had difficulty explaining these results, as they did not seem to follow a pattern. Notably, the previous study utilized KieroSeal^®^, whereas our study utilized GapSeal^®^, which has a different composition. Because GapSeal^®^ never hardens, it is not associated with shrinkage. In contrast, KieroSeal^®^ has a polymerization time of 5 min (according to the authors), and its composition is predominantly siloxane-based, which causes dimensional changes during polymerization.

Although we did not find any studies evaluating the effect of PTFE tape on the preload, we found one study testing PTFE materials of different thicknesses; however, the PTFE was applied to the screw thread via thermal spraying instead of using a tape. The authors of that study recorded the screw joint clamping force (preload), whereas this study recorded the preload on the screw [24]. Their results also differed from ours because the PTFE group in their study showed a higher preload compared to those associated with the other groups. As mentioned previously, the test methodology used by Chen et al. [24] is very different from the one used in this study; hence, it is not possible to compare the results.

Another study [25] tested different abutment screw coating materials (titanium carbide, titanium carbo-nitride, PTFE, and Parylene) and found a decrease in the friction coefficient and an increase in the preload for a given tightening torque; however, it is difficult to compare the results with ours because of the differences in the methodology used in both studies.

### 4.2. RTV Analyses

The RTV of each sample was recorded after the preload on the screw was registered. The results showed statistically significant differences among all groups, with the GC group showing the highest RTV, followed by the GC group, while the GP group showed the lowest results. Thus, the null hypothesis was rejected.

Other authors have reported results consistent with ours [20,26]. On the other hand, Ozdiler et al. [27] reported a higher percentage loss of RTV in the sealing silicone gel group than in the other groups, with a significant difference. Notably, the authors only registered the RTV after cyclic loading. They also explained that their results might be related to the setting contraction behavior and dimensional stability of silicone materials, which are affected by changes in temperature and humidity. As previously explained, the application of a sealing gel might reduce the coefficient of friction and consequently increase the preload. As the preload increases, more torque is required to remove the screw; therefore, theoretically, the chances of the screw loosening will be lower. Yu et al. [20] used the same sealing silicone gel used in our study and subjected the samples to mechanical cycling; the results with the sealing gel were favorable, suggesting that the high viscosity of the silicone gel could act as a protective layer against external loading impact.

To our knowledge, only three studies have evaluated the effect of PTFE on the RTV. Chen et al. [24] tested two different thicknesses of a PTFE coating material (30 and 60 µm), using thermal spraying instead of tape. Although they reported better RTV results with the 30 µm thickness than in the control group without any coating, significant differences were only reported with the 60 μm thick coating. These results do not corroborate our results, which could be attributed to the difference in the application of the PTFE. Our study used tape, whereas theirs sent the screws to different companies for coating. Elias et al. [25] also tested the effect of PTFE on the RTV, and their results do not corroborate our findings. They tested four different torques and only found statistically significant differences in the groups with lower torques (20 N cm and 30 N cm). However, in the group tightened to 20 N cm, the PTFE had a negative influence, resulting in a very low RTV, and in the group tightened to 30 N cm, the PTFE had a positive influence, resulting in a significantly high RTV. Although Felix et al. [21] used the same PTFE tape as used in our study, their results are contradictory to ours. Our study reported the lowest results in the GP group, whereas theirs reported a significantly higher RTV in the PTFE group than in the control group without any coating. These differences could be explained by the different thicknesses used in both studies. We used a 75 μm thick tape and wound the screw twice, whereas they used a 100 μm thick tape and wound it thrice, thus achieving 300 μm of thickness. In addition, they only registered the RTV after cyclic loading.

### 4.3. Relationship between Preload and RTV

Additionally, by comparing the preload value to the RTV, we found a statistically significant relationship in the GG group, suggesting that the preload positively influences the RTV. Data in the literature on the effect of silicone sealants on the preload or RTV are limited. Other studies have shown a relationship between the preload and the RTV, suggesting that a higher preload will result in a higher RTV [9,25].

A limitation of the present study is the in-laboratory setting of the study, which may not necessarily replicate the intraoral environment. For future research, samples should be submitted to a chewing test machine to simulate at least one year of mastication forces before testing in patients. We also experienced difficulty in applying the PTFE tape, which might not be uniformly distributed around the prosthetic screw, thus compromising the results. It could therefore be helpful to evaluate the PTFE’s distribution using a stereomicroscope with magnification prior to testing.

## 5. Conclusions

Within the limitations of this study, it can be concluded that the application of PTFE tape on the prosthetic screw significantly reduces the preload while the application of the sealing gel (Gapseal^®^) has no effect on the preload.

The RTV results differed significantly between the groups. The RTV was the highest in the group using the sealing gel (Gapseal^®^) and the lowest in the group using PTFE. Therefore, use of this sealing gel should be considered to prevent the loosening of the abutment screw, while the PTFE should not be used.

The high preload in the group using the sealing gel (Gapseal^®^) increased the RTV, suggesting that a higher preload will lead to a higher RTV.

## Figures and Tables

**Table 1:** ANOVAs and ANCOVA for unadjusted and adjusted comparisons of preload and RTVs by groups.

	Control (*n* = 15) M (SD)	Gapseal (*n* = 15) M (SD)	PTFE (*n* = 15) M (SD)	ANOVA
Preload	30.41 (1.04)	29.86 (1.39)	28.88 (1.07)	F_(2,42)_ = 6.45 (*p* = 0.004), η^2^ = 0.23
RTV	26.59 (1.25)	28.67 (1.47)	16.16 (2.89)	F_(2,42)_ = 167.00 (*p* < 0.001), η^2^ = 0.83
	adjM (SE)	adjM (SE)	adjM (SE)	ANCOVA
RTV	26.40 (0.59)	28.70 (0.49)	17.20 (0.63)	Group: F_(2,41)_ = 177.04 (*p* < 0.001), η^2^_p_ = 0.90 Preload: F_(1,41)_ = 3.53 (*p* = 0.068), η^2^_p_ = 0.08

ANCOVA adjusted to preload; results presented as means (M) and standard deviations (SD) for univariate analysis (ANOVAs) and adjusted means (adjM) and standard errors (SE); η^2^, eta squared; η^2^_p_, partial eta squared.

## Data Availability

Data are contained within the article.
